# Child-, Family-, and Community-Level Facilitators for Promoting Oral Health Practices among Indigenous Children

**DOI:** 10.3390/ijerph19031150

**Published:** 2022-01-20

**Authors:** Brianna F. Poirier, Joanne Hedges, Lisa G. Smithers, Megan Moskos, Lisa M. Jamieson

**Affiliations:** 1 Australian Research Centre for Population Oral Health, Adelaide Dental School, University of Adelaide, Adelaide 5000, Australia; Joanne.Hedges@adelaide.edu.au (J.H.); Lisa.jamieson@adelaide.edu.au (L.M.J.); 2 School of Public Health and the Robinson Research Institute, University of Adelaide, Adelaide 5000, Australia; lsmithers@uow.edu.au; 3 School of Health and Society, University of Wollongong, Wollongong 2522, Australia; 4 Future of Employment and Skills Research Centre, School of Economic and Public Policy, Faculty of the Professions, University of Adelaide, Adelaide 5000, Australia; Megan.moskos@adelaide.edu.au

**Keywords:** Indigenous peoples, oral health, dental caries, public health dentistry, motivational interviewing

## Abstract

Despite the preventive nature of oral diseases and their significance for general wellbeing, poor oral health is highly prevalent and has unfavourable ramifications for children around the world. Indigenous children in Australia experience disproportionate rates of early childhood caries compared to their non-Indigenous counterparts. Therefore, this paper aims to collate parental experiences and generate an understanding of facilitators for Indigenous childhood oral health. This project aggregated stories from parents of Indigenous children across South Australia who were participants in an early childhood caries-prevention trial. This paper explores facilitators for establishing oral health and nutrition behaviours for Indigenous children under the age of three through reflexive thematic analysis. Fisher-Owens’ conceptual model for influences on children’s oral health is utilised as a framework for thematic findings. Child-level facilitators include oral hygiene routines and regular water consumption. Family-level facilitators include familial ties, importance of knowledge, and positive oral health beliefs. Community-level facilitators include generational teaching, helpful community resources, and holistic health care. Recommendations from findings include the following: exploration of Indigenous health workers and elder participation in oral health initiatives; inclusion of Indigenous community representatives in mainstream oral health discussions; and incorporation of child-level, family-level, and community-level facilitators to increase support for efficacious oral health programs.

## 1. Introduction

Childhood oral health is fundamental to overall health and supports essential functioning, enables painless eating, speaking, socialising, and smiling, and strengthens quality of life and self-esteem [1,2,3]. The impacts of poor oral health in children are well documented [4,5,6,7]. Presence of early childhood caries (ECC) is an indicator of social disadvantage and an early measure of deprivation-related poor health [8]. Despite the significance of oral health to general wellbeing, ECC is the most prevalent chronic disease globally, affecting 60–90% of children [1,9]. The World Health Organisation has called for widespread community efforts and government support to address the prevalence of ECC, especially in disadvantaged communities [10,11].

Across unique geographic locations, cultures, and histories, Indigenous peoples experience strikingly similar poor oral health outcomes [12], related to the impacts of colonisation, assimilation, discrimination, and marginalisation [13,14]. The cultures of Aboriginal and Torres Strait Islander peoples (respectfully, subsequently referred to as “Indigenous”) in Australia are among the oldest thriving in the world [15]; prior to colonial invasion, there were at least 260 distinct language groups with unique histories, cultures, and spiritual traditions across the continent [16]. With colonisation in Australia came Indigenous suffering through removal from traditional lands, restriction of language use, child removal, ecological destruction, marriage regulation, and suppression of participation in cultural activities [13,17]. Factors contributing to oral disease for Indigenous peoples in Australia are rooted in the social devastation caused by colonisation [18]. The social determinants related to colonisation are compounded by changes to diets and lifestyles and underscored by a lack of understanding and prioritisation of Indigenous cultural values in health care [17,19]. Despite the legacy of colonisation and the preservation of colonial structures in societal and health systems, Indigenous peoples in Australia continue to be resilient [20].

Dental public health efforts to improve childhood oral health in Australia are not reflected in Indigenous children, as evidenced by high oral disease burden, high rates of dental surgery requiring anaesthesia, and low rates of dental visits [21,22,23,24,25]. Prevention of ECC has historically depended upon individual adherence to behavioural messages, such as tooth brushing and reducing sugar consumption; however, it is increasingly acknowledged that messaging alone rarely results in sustained behaviour change [26]. The Australian government has acknowledged the disadvantage of Indigenous communities and the strong evidence equating Indigenous health disparities to the overwhelming impacts of trauma and marginalisation, as results of government policy [18]. Improving Indigenous childhood oral health is a mandate of the Australian government’s oral health plan [27]. The 2012–2013 Australian Aboriginal and Torres Strait Islander Health Survey reported that 80% of individuals aged 2 years and over who identified needing to go to the dentist did, approximately half the respondents brushed their teeth twice daily on a regular basis, and only 14% had never seen a dentist [28]. Through the development of their Aboriginal Oral Health Program, the South Australian Dental Service has observed a significant increase in the number of Indigenous children accessing dental care over the past 10 years, from 2895 children in 2006 to 4717 in 2016 [29]. 

It is imperative that the exploration of Indigenous children’s oral health is framed by Indigenous experiences, beyond the traditional biomedical focus of oral health [30,31]. The greatest impact on childhood oral health practices is caregiver influence, highlighting the importance of family participation in ECC prevention [32,33,34]. Prioritising parent perspectives helps generate an understanding of the factors that facilitate oral health for children [35], which can include connection to family, community, country, culture, identity, and spirituality [30]. It is widely acknowledged that oral health disparities are driven by multifaceted circumstances [13,36,37]; as such, consideration for the diverse factors that impact overall child wellbeing is necessary. Qualitative research provides the flexibility that is required to capture factors important to participants, but few qualitative works explore parental perceptions of the range of factors that improve oral health for children and instead focus more specifically on tooth brushing or oral health attitudes [38,39,40,41]. 

Dominant discourse in Australia centralises notions of failure on the part of Indigenous communities to thrive. Resilience literature underscores the importance of highlighting a more holistic narrative of Indigenous wellbeing, challenging stereotypes, and substantiating the immense individual and collective strength in defiance of continuous obstacles [42,43]. While disease outcomes remain highly disproportionate for Indigenous peoples across the country and in South Australia, focusing on improvements and the positive outcomes of oral health can help motivate, rather than discourage, Indigenous communities [44,45]. To date, the majority of research in Indigenous health has employed a deficit approach [46]. This paper aims to utilise a strength-based approach [46] through the prioritisation of Indigenous voices, the collation of parent stories of strength, and the exploration of parent-identified facilitators for Indigenous childhood oral health. 

## 2. Materials and Methods

### 2.1. Study Design

This project is part of a randomised control trial of an ECC intervention that prioritised collaboration and partnership with Indigenous communities and families across South Australia (Trial Registration: Australian New Zealand Clinical Trial Registry ACTRN12611000111976). The findings from this analysis have been reported in alignment with the Consolidated Criteria for Reporting Qualitative Research (Supplementary Material S1) [47] and the Consolidated Standards of Reporting Trials (Supplementary Material S2) [48]. The protocol [49], primary quantitative results [50], and cohort profile [51] have been published. The trial enrolled 448 women, pregnant with Indigenous children at baseline, who were randomly allocated to intervention or control (delayed intervention) groups. The intervention had four elements, including: (1) dental care provision for mothers during pregnancy; (2) fluoride varnish application for children; (3) anticipatory guidance; (4) motivational interviewing (MI). The findings in this paper are from the MI component; MI is an empathetic behavioural support method that encourages identification, exploration, and resolution of obstacles to change behaviours [52]. This psychotherapy intervention is rooted in the notion that intrinsic motivation increases the likelihood of behaviour change, and that knowledge alone is insufficient to elicit behaviour change. Notably, MI respects the cultural values of Indigenous peoples, such as the oral traditions of yarning [53] and self-determination, yielding a more holistic understanding of a given issue [54,55]. MI has been successfully utilised to elicit oral health behaviour change for parents and children [56,57].

In this trial, MI occurred at baseline and when the child was aged 6, 12, and 18 months for the intervention group. The respective directives for each motivational interview were: (1) supporting dental care during pregnancy; (2) discussing the significance of non-cariogenic drinks and foods for children; (3) emphasising the role of fluoride in ECC prevention; (4) encouraging the child’s first dental visit. The control group had interviews when the child was aged 24, 30, and 36 months; the first interview combined directives (1) and (2). Due to the patient focus of MI, there were no set questions for each session; however, guides were developed in partnership with MI training facilitators and included prompts, key messages, and activity guidelines for each interview. Each session guide was piloted by interviewers prior to trial commencement. These data were collected from February 2009 to May 2013. All interviews were audio recorded and transcribed verbatim at the end of data collection. Interviews varied in length from 20 to 90 min.

### 2.2. Ethical Approval 

The project received ethical approval from the Aboriginal Health Council of South Australia (04-09-362) and the University of Adelaide Human Research Ethics Committee (H-057-2010). Procedures for confidentiality were adhered to and informed written consent was obtained from all participants.

### 2.3. Participants and Sampling 

Interviews were purposively sampled for this analysis due to the variation in MI delivery and fidelity scores across the four trained staff involved in the interview component. Successful MI is contingent on interventionist competency in eliciting statements of self-motivation from participants [58]. Fidelity assessment of MI was conducted to measure the extent to which the intervention was performed as intended and to ensure methodologic rigour in this trial [59,60]. All interviews included in this analysis were completed by the single staff member with the highest MI fidelity score—a senior Indigenous researcher who worked to establish reciprocal and trusting relationships with participants. These interviews had the richest conversations and were more comparable with one another, providing the highest quality data to answer the research question. 

### 2.4. Data Analysis 

Reflexive thematic analysis embraces researcher subjectivity and acknowledges that researchers are embedded in project design and analysis, and that findings are inescapably influenced by researcher interpretation [61]. As such, it is critical to acknowledge the assumptions and perspectives one brings to research. The primary author is a non-Indigenous researcher from Canada who has been working with the same communities and Aboriginal Health Workers (AHW) involved in this trial, actively taking opportunities to enhance her contextual and cultural understandings of the environment in which the experiences highlighted through this work exist. Both the senior Indigenous researcher (J.H.), who conducted the interviews, and the trial’s primary investigator (L.J.) have been working with Indigenous communities in SA for over a decade. 

Reflexive thematic analysis, as detailed by Braun and Clarke, guided this analysis [61,62,63]. Significant time was spent in an initial phase of reading, listening, and reviewing each interview multiple times to ensure researcher closeness with the data [64]. During this time, the primary author noted initial ideas from each interview. Extensive discussions of data and initial thoughts took place between the primary author, the senior Indigenous researcher (J.H.), and the primary investigator (L.J.) prior to thematic analysis. Following the data immersion phase, the primary author inductively coded the data line by line in NVivo 12 software (QSR International Pty Ltd. Version 12.6.1, Melbourne, Australia) to identify features of the data relevant to the research question; the entire dataset was given full consideration. After all transcripts had been coded, the primary author reviewed all of the codes and similar codes were aggregated into themes. At this stage, an initial thematic map was created to help the researchers consider the relationships between themes, as suggested by Braun and Clarke [64]. Any themes that did not have sufficient data to support them were discarded. Refinement of themes happened in relation to the thematic map, as well as the entire dataset, to ensure representation of all interviews; any final coding needed to ensure data coverage was performed at this time. Next, themes were clearly defined and labelled, not only in relation to specific findings but also in relation to the overall story of the dataset. Finally, during the report production phase, illustrations from transcripts were chosen that exemplify the nature of each theme. 

### 2.5. Fisher-Owens’ Conceptual Model 

Due to the immersion of oral health behaviours in complex daily life [65], ECC prevention cannot singularly focus on individual behaviour or biology, but must also consider the wider psychosocial, environmental, and corporate determinants of oral health [11]. These determinants are considered in Fisher-Owens and colleagues’ comprehensive conceptual model, where individual, family, and community influences on children’s oral health are considered [11]. While atypical in reflexive thematic analyses [61], a modified version of the Fisher-Owens and colleagues’ model [11] was utilised as a framework for this analysis because of the opportunity it provided to consider a more extensive understanding of the diverse environments in which oral health exists. Each level of the Fisher-Owens and colleagues’ model considers five domains: genetics and biology, social environment, physical environment, health-influencing behaviours, and medical and dental care [11]. This potential use of this model was considered after thematic development and prior to report production due to its alignment with the patterns identified in the data. Some domains within each level did not relate to the findings; therefore, a modified framework was employed that considered social environment, physical environment, and health-influencing behaviours. 

### 2.6. Consideration of Socioeconomic Positions 

Factors of employment, income, and education relate to broader socioeconomic and historic environments and influence oral disease, as well as utilisation of oral health services for Indigenous peoples in Australia [2,66,67,68]. Participants were classified according to socioeconomic position (SEP) to provide context for views expressed during the motivational interviews, and because approximately 34% of the health gap between Indigenous and non-Indigenous Australians is attributable to social determinants of health [69]. SEP considers diverse elements of economic and social wellbeing in relation to class position [70]. SEP influences oral health impacts through various mechanisms due to its relationship with resource access, disease consequence, and ability to benefit from new knowledge [71]. Utilising five measures—including maternal education, health care card status, car ownership, ability to pay a AUD 100 dental bill, and regional measures of socioeconomic advantage and disadvantage—families were classified as either high SEP or low SEP. All measures were dichotomised and families that had at least three of the possible five high SEP factors were categorised as such, any families with less than three were categorised as low SEP (Table 1).

Eligibility for public dental services in Australia is defined by Centrelink, a Commonwealth Department of Human Services agency, which includes a health care card program that assists cardholders with medical fees [72]. Health care card status is contingent on low family income, among other criteria; therefore, card status was employed as a proxy indicator of family income for SEP categorisation, rather than maternal income [72]. The index of relative socioeconomic advantage and disadvantage (IRSAD), based on family postal area from the Australian Bureau of Statistics 2011 census of population and housing, was included as a measure of environmental impact. The IRSAD considers both advantage and disadvantage, encapsulating information about social and economic environments of households within an area [73]. Further, maternal education is related to food knowledge and food choice, which directly impacts child nutrition [74]. 

## 3. Results

**Figure 1:**
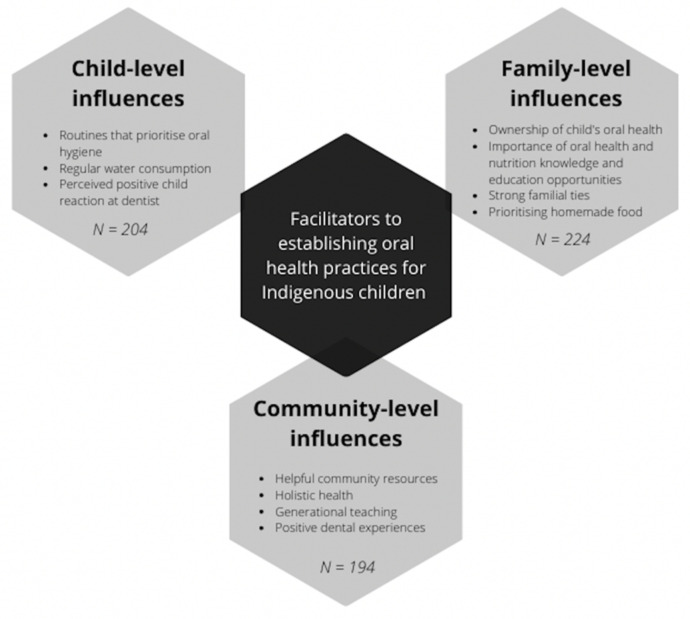
Conceptual model of facilitators to establishing oral health practices for Indigenous children in South Australia.

Findings from 327 interviews with 226 carers from Indigenous communities across South Australia are presented in the order of the highest to the lowest number of participants that discussed themes within each of the following categories: family-level influences (*N* = 224), child-level influences (*N* = 204), and community-level influences (*N* = 194) (Figure 1). In this sample, 28.9% of families were categorised as high SEP, as determined by maternal education, health care card status, ability to pay a AUD 100 dental bill, car ownership, and IRSAD by postal area (Table 2). 

### 3.1. Family-Level Influences

#### 3.1.1. Ownership of Child’s Oral Health 

Parent ownership of their child’s oral health facilitated personal responsibility for parents who identified oral health as a central component of their role as a parent; when discussing why one father ensures his child brushes his teeth every day, he replied: *“Because it’s my duty, it’s my duty… I’ve got to step up to the plate, don’t I?” (P20; Low SEP father).* Parent ownership of their child’s oral health related to parent initiative and determination. Initiative and determination facilitated oral health for children because parents took steps to learn new information, which prepared them for their child’s evolving oral health needs, such as wiping baby teeth after feeds, making dental appointments, and introducing a toothbrush. Rationale for wanting to learn more was succinctly explained by one mother, as follows: *“Yes, always wanting to learn more about, you know, the nutrition and the wellbeing and how what I do as a mum can affect that with my kids” (P37; High SEP mother).* Even within motivational interviews, parents exemplified a desire to take action; once learning something new, parents wanted to implement their knowledge as quickly as possible and began brainstorming ways in which they could:

*“I probably will give less juice and yes, wow, yes, maybe try and rethink some choices like, because … when you go out to places and you get kids meal deals and various things, you get the juice, like you pick that one, but I’ll pick differently in future”.*

(P161; High SEP mother)

Ownership of child’s health related to an appreciation of the importance of the dentist and the life-long importance of teeth. These beliefs facilitated oral health for children because these notions underscored the importance of oral health for parents and elevated priority of oral health related behaviours and routines, *“You know, you only get two sets of teeth in your life. I don’t want her to have dentures before she’s 20, you know” (P10; Low SEP mother).* Parents discussed learning from previous experiences as a facilitator to prioritising the importance of their children’s oral health. Both personal experiences and observation of friends and families’ experiences taught parents how individual behaviours can impact oral health outcomes; this notion held true for both positive and negative experiences. For example, some parents were very proud of their healthy teeth; therefore, they wanted the same outcome for their children. Meanwhile, other parents identified personal oral health problems and were adamant to avoid this outcome for their children:

*“Well like I said, I’ve got [dental caries] in my mouth, so I don’t want my kids to have them, because they’re horrible. I’ve got yucky teeth, and that’s from growing up where I didn’t brush my teeth because nobody told me I had to brush my teeth. I just don’t want that [for them] because my teeth are all falling out and it’s horrible”.*

(P124; High SEP mother)

#### 3.1.2. Oral Health and Nutrition Knowledge and Education Opportunities

The importance of oral health and nutrition knowledge and education opportunities were frequently discussed: *“(Interviewer): So, knowledge is power? (Interviewee): Yes. I wish we had a lot more” (P18; Low SEP mother).* Those with knowledge shared stories about going out of their way to share their understanding with others and strengthen their family health. Accessibility to education and correct knowledge varied among participants. Many parents cited nutrition education as a result of a health condition, such as diabetes mellitus, or previous dieting. In particular, the ability to read nutrition labels and knowledge about the impact sugar has on one’s health resulted from disease-related education: *“That’s why I’ve been really cautious on what he does have and don’t really agree with him having too many sweet things at all just for like family diabetes issues as well. Not only like teeth” (P21; Low SEP mother).* Regardless of the source of knowledge, parents agreed that correct knowledge and access to education was one of the most important things to ensuring oral health and general wellbeing.

*“Like I said to my doctor … it’s being educated. When you have that education and understanding, you can change things in your life. Where beforehand I didn’t have that understanding of what foods are good and some foods that you’d always considered good, turn into sugars and you eat them and all that, so education [is the most important] I think”.*

(P20; Low SEP mother)

Associations between individual health behaviours and potential outcomes heavily increased awareness for participants and facilitated healthy behaviours. Associations included the relationships between bottle, fluoride, sugar, water, general health, and oral health. Many parents cited correct information shared during initial motivational interviews in subsequent sessions:

*“Yes, because you told me about that, so that’s just made me aware of, you know, letting him sleep with a bottle, because the milk just lays on their teeth, and see I never knew that… because the first boy, he was on the titty all the time, and then when he come off, he was just on water, see, so, he had no problems with his teeth, but [this child] just wanted bottle all the time”.*

(P33; Low SEP mother)

Parents were quick to identify unhealthy practices in other parents, in particular with regard to giving children sugar and dental surgery, justifying their judgment by assessing the lack of prevention efforts other parents made. These comparisons facilitated oral health of children because parents were hyper-aware of their own actions in comparison with those they observed in others:

*“Just sad, you know, kids going to have to go under a local anaesthetic to get their teeth out…the parents should, you know, should know better, not to… Like I said, they need tough love, you know, not to give in straight away. A lot of kids do cry to get their way, but that’s where I just, no, I’m not going to give in to you, you can cry, chuck a tantrum all you want”.*

(P64; Low SEP mother)

#### 3.1.3. Strong Familial Ties 

Dynamic and supportive relationships in the home, between parents and children, as well as between parents created an environment conducive to the establishment of oral health practices for children, “*We all go. My whole family goes. If [my sons] got an appointment my whole family will go” (P8; Low SEP mother).* Parents appreciated when they were able to communicate expectations with their partners and they were supported in attaining oral health goals. Familial ties provided parents with readily available help and unwavering support: *“Me and [my wife’s] brother and sister, we all work together, all as one. Yes, we all work as one. And we just all go together, support one another” (P4; Low SEP father).* This support extended to oral health practices; parents expressed deep gratitude for family members that respected or shared their values with regard to their child’s oral health. Family members who assisted with appointment transportation, maintained oral health habits, and encouraged reduced sugar consumption facilitated oral health for children. Some parents even mentioned that once they had clarified the impact sugar can have on children’s teeth, grandparents began giving toys and games to their grandchildren, instead of lollies. Family support was discussed for many as *“just how it is”* and something that was important to teach to children:

*“Family have helped in getting us to where we are now. So it’s like looking after babies and everything like that. It is like a necessity basically that we teach our children and in that way they can teach their children and so on, and so on and so on. So it does play a big role in healthy teeth”.*

(P18; High SEP mother)

Strong familial ties related to positive household role models, both in terms of parent influence and sibling influence. Siblings were primarily described as aiding with tooth brushing and dental visits as younger children would follow their example. Parents discussed limiting their own sugar and changing their dietary patterns for the benefit of their children’s health:

*“Well even with me, I’ve gone on a diet myself, so I’ve cut out the cool drinks and the juice… so I’ve been more on the water myself. So maybe the kids are seeing that too, because all I’m doing is drinking water, so they’ll end up grabbing my water bottle and, you know, if it’s got a pull top then they’re drinking that. So that’s good”.*

(P110; High SEP mother)

#### 3.1.4. Prioritising Homemade Foods 

Parental effort to establish oral health and nutrition behaviours included the prioritisation of bush tucker at home and limiting sugar or processed food consumption. Many participants reflected on the influence their parents and grandparents had on their eating habits: *“We rarely had packaged stuff growing up, I think…My mum’s not so big on ingredients but just like growing up, we never had pre-packaged stuff. It was always healthy whole food” (P2; Low SEP mother).* Parents who made most of their food at home stressed the importance of knowing exactly what they are giving their child as well as a decreased reliance on processed foods, often high in sugar. The ability to read and understand a nutrition label, as well as having sufficient time for shopping, further facilitated healthy food choices for parents. Some parents discussed the impact sugar had on their children’s health and behaviour and made efforts to limit sugar consumption by watering down SSB and treating with something other than sugar, such as toys or stickers.

*“I don’t give into lollies, I don’t give into that. They do have their odd ice-cream every now and then, but not every time…Yes, so it’s not always about lollies or soft drinks, or things like that. I give them treats in other ways, like buying them a toy, or you know going to the street, go to the beach, going to the playground, and things like that”.*

(P64; Low SEP mother)

### 3.2. Child-Level Influences 

#### 3.2.1. Routines That Prioritise Oral Hygiene 

Parents discussed routines as fundamental to establishing oral health-promoting behaviours for their children at a young age. Routines were deemed important for parents to prepare their children for the future, *“They’re not looking after baby teeth they’re not likely to look after adult teeth, you’ve got to start the routine early” (P20; Low SEP mother).* Parents identified routines as a mechanism to avoid unnecessary dental procedures, potentially saving money. Routines were also discussed in terms of decreased child reliance on night-time bottle feeds. Older siblings were mentioned as helpful in decreasing bottle use because their example helped children *“grow up a lot quicker.”* Enjoyable and fun tooth brushing enticed children to adhere to routines; strategies mentioned by parents included colourful and themed brushes, using phone applications with brushing songs and dances, enlisting the help of older siblings, praise, or the use of a sticker chart.

*“I think it’s just the toothbrush I bought for him, it’s a little Batman light up one, it’s lights up for [the time] that you have to brush for and as soon as it stops you stop brushing, yes, so I think that’s what it is, the toothbrush, he likes it”.*

(P70; Low SEP mother)

#### 3.2.2. Regular Water Consumption

For many children, water was initially introduced on hot days to quench thirst, or when children were sick. Many parents identified the importance of water for their child’s health, especially in terms of reducing sugar-sweetened beverage (SSB) consumption, and tried to increase water availability for their children. Parents believed that water was important for the entire body: “*Well I know it can strengthen your teeth and your gums… It helps your liver. Everything like that. It’s more or less a cleanser for your body, your whole body” (P17; Low SEP mother)*. Some parents struggled with resistance to water from children and noted if they had prioritised water consumption earlier, the process would have been made easier.

*“He’s been looking for the water … we’ve got one of those water fountains, you know, that you can go on press and it’ll come out. We’ve been finding water on the floor, because he’s going and standing underneath it and drinking it. His dad growled him, and I said don’t growl at him, you encourage him, he’s drinking water, you know, that’s the best thing for him”.*

(P10; High SEP mother)

#### 3.2.3. Perceived Positive Child Reaction at The Dentist 

Perceived positive child reaction at the dentist was mentioned by parents as a facilitator to attend child’s first dental appointment. This perception was grounded in children’s previous health care experiences, reactions, and behaviours. Some parents shared stories about previous positive experiences with other healthcare professionals: *“He sits really still and he’s very co-operative [at the doctor]… it’s just new to him and when it’s new to him he’s just like really quiet and observing and he doesn’t run amok” (P175; High SEP mother).* Positive parent perceptions eased worries or apprehensions, facilitating dental appointment attendance.

*“Well he just loves being the centre of attention. I guess being the second child he always… The first ones always try to get all the attention and I think him being up in that chair with the dentist and everyone focused on him, I think he will like it”.*

(P57; High SEP mother)

### 3.3. Community-Level Influences 

#### 3.3.1. Helpful Community Resources 

The majority of community level factors that parents discussed as facilitating improved oral health for children were grounded in relationships with others, such as helpful community resources and sharing oral health information with others. Helpful community resources mentioned by parents included the Aboriginal Community Controlled Health Services (ACCHS), AHW, midwives, dental services, hospitals, parenting classes, Mum groups, online sources, friends, and school programs. In particular, home visits from the Child and Family Health Service (CaFHS) nurses were cited as extremely accessible and useful, with many parents discussing the approachability of this service and emphasising their ability to discuss any concerns or questions during visits. Additionally, some parents discussed the usefulness of various community services in bridging the gap between community members and health services, especially with regard to improving the accessibility of dental services for community members. Some communities provided transportation to the dentist or had a community liaison to provide support for parents before, during, and after appointments.

*“If you want your dental work … [the support person] can go with you and pick you up and take you to a dental clinic… You know, for the fella’s that have got really bad teeth … I suppose she explains to the dentist beforehand that, you know, like be prepared more or less, like don’t say this is this… You know, don’t let these fella’s walk in and be like oh my God you didn’t brush your teeth, because obviously they haven’t… She prepares them so you don’t feel bad about not looking after your teeth”.*

(P19; Low SEP mother)

School programs were discussed as helpful by parents, not only in terms of dental services provided at schools but regarding food school policies, which included the provision of nutrition information and the restriction of certain items, such as packaged foods and SSB. Specifically, water only policies in kindy and daily tooth brushing at day care helped parents strengthen existing efforts at home. Some parents talked about having certain processed snacks, typically ones high in sugar, sent back home because they did not follow nutrition guidelines, which influenced parent shopping:

*“[The school] keep[s] carrying on about the packaged foods and things so that’s helped us with our snacks and things like that I’ll get other things… because [some snacks have] higher sugars and higher salts… And even though they might be labelled school snacks not necessarily healthy so being quite conscious since we’ve come to this school it’s helped to you know, open our eyes up a bit more. As in the packaging’s and yes, so we tend to read and they’ll be like but it’s in the school snack aisle. I’m like yes but look what it says here in the little bar of how high the sugar actually is in this and the school’s going to go you can’t eat that”.*

(P40; Low SEP mother)

#### 3.3.2. Holistic Health 

The prioritisation of holistic health within healthcare systems was identified as a facilitator by participants. One mother discussed the importance of the whole body and variety of life factors that are considered when she utilises ACCHS, in comparison with the singular treatments she has experienced with other health professionals. Another Mum described feeling lost after having her teeth removed: *“I was attached to my teeth, the teeth were attached to me” (P46; Low SEP mother).* Other parents mentioned the importance of the whole mind, body, and spirit when considering oral health efforts, especially the interconnected relationships between oral health and child self-esteem. Participants heavily valued the holistic approach to healthcare accessible through ACCHS, as well as the trusting and supportive relationships that AHW prioritised with their patients.

*“I go [to the Aboriginal health workers] a lot. Like, even if it’s got nothing to do with health, when I need, just, to chat about something, I will go there… So they’re good not just for health but for everything, whereas, when you go to the doctors it’s not really the same”.*

(P26; Low SEP mother)

#### 3.3.3. Generational Teaching 

Generational teaching heavily influenced parental oral health beliefs and in turn, children oral health habits, including nutrition, teeth cleaning, breastfeeding, dental visits, water consumption, and the conceptualisation of the importance of teeth. Parents established the importance of elders and ancestors in their oral health journey: *“Always listen to the elder ones. They always know the best” (P21; Low SEP mother).* Many participants talked about the importance of maintaining generational teaching for family health in generations to come. An underlying notion within the concept of generational teaching was the effort to preserve connection and longevity that has been disrupted for generations:

*“It was good that my mum and dad were so concerned with our oral health, you know, that’s why I’m so with my kids…Yes, well, I want our line to stay strong. Do you know what I mean? For generations my family was always wiped out, and then it was only in my mum’s generation that we’ve just sort of started to come back together”.*

(P2; High SEP mother)

Generational teaching also related to experiences of knowing family or community members with poor oral health. Parent observations of family members with poor oral health influenced their oral health understanding and was often a learning experience for what not to do with their own children. Many parents mentioned siblings or cousins who relied on sugary drinks in baby bottles. Some participants used older family members with rotten or missing teeth to encourage oral health with their children:

*“[The kids] understand that, you know, you’ve got to look after their teeth otherwise… Because my Dads got all falsies and I try and explain to them, oh you’ll have no teeth when you’re old. You’ll look like your Poppa”.*

(P63; Low SEP mother)

#### 3.3.4. Positive Dental Experiences 

Positive dental experiences facilitated recurring dental visits, central to the establishment of oral health. Past parental experiences, as well as experiences with children, impacted frequency of dental visits: *“I like the dentist, I want a good memory of the dentist. So I want my kids to have good memories of the dentist so that they look forward to going to the dentist in the future” (P91; Low SEP mother).* Some parents discussed having older siblings help ease early dental experiences and others noted how much their children enjoyed receiving stickers and new toothbrushes at the end of appointments. Many parents utilised the dentist as a source of reassurance that they were on track with their child’s oral health: “*I’ve actually got in mind now I want to make a dentist appointment so I can see how [his] teeth are going. I’m quite curious. Then I can get some more information too” (P18; High SEP mother).* Parents identified the importance of being comfortable with the dentist as a facilitator to attending appointments, many wanting a dentist that regularly works with children:

*“I know, you know, [the dentist] can detect things that I can’t see and catching anything, you know, at an early stage would be good. And it gets her used to it too, you know, she won’t be scared of the dentist when she goes in kinder or school or whatever. She’ll know that going to the dentist is a good thing not a scary thing like other kids”.*

(P131; Low SEP mother)

## 4. Discussion

This research sought to collate parent experiences and generate an understanding of facilitators for improved Indigenous childhood oral health in South Australia. The results emphasise the importance of considering facilitators beyond an individual child to include family and community-level facilitators. Key results from this analysis include familial ties, learning from previous experiences, positive oral health beliefs, generational teaching, helpful community resources, and holistic health care as facilitators for parents in this project. Oral health interventions have had varying success in Indigenous communities due to fragmentation from other areas of health, counterintuitive to the multifactorial nature of ECC [13,36,75,76]. The findings presented in the conceptual model align with the previous literature, in that they emphasise the necessity of robust oral health prevention efforts for Indigenous communities that consider overall wellbeing in conjunction with biomedical measures. Through prioritisation of Indigenous voices, this paper highlights the ways that research could drive future public health policy agendas—strengthening the parent-identified pathways to good oral health for their children. 

### 4.1. Family-Level Influences 

Parental knowledge was critical to establishing oral hygiene and healthy nutrition practices for families in this project. Findings highlighted that some mothers had developed food literacy skills as a result of dieting or education related to a health condition, such as diabetes, rather than through preventive oral health programming. Parent and carer nutrition knowledge impacts child oral health due to food provision responsibilities and the influence of dietary patterns established early in life on dietary behaviours later in life [77,78]. Through prioritisation of bush tucker and foods made in the home, parents in this study limited reliance on processed and sugary foods. Approximately half of the families in this study live in non-metropolitan areas where food costs have remained significantly higher than in metropolitan areas for over 20 years [79]. Particularly for individuals of lower socioeconomic position, this seriously impacts their ability to decrease their reliance on cheaper food options, which are often ultra-processed and of low nutritional value [80]. 

Parents’ oral health beliefs and understanding of consequences related to poor oral health have previously been identified as facilitators to maintaining oral health practices for children [81]; similarly, formation of associations between parent actions and health outcomes during motivational interviews generated a deeper understanding of consequences for parents and a readiness to take action in our trial. Related to parent knowledge was parent judgment or observation of other children’s teeth and subsequent comparisons related to their own child’s oral health. This pattern of parent observation has been noted elsewhere [81,82]. While learning from personal mistakes or observing others’ mistakes can teach parents what not to do, it does not necessarily teach them the necessities for oral health in children. Given the centrality of parent knowledge to child oral health, as well as the desire from parents of Indigenous children here, in Queensland [83] and in Western Australia [82], for more practical advice and education, there may be a gap in oral health education provision and community needs. 

### 4.2. Child-Level Influences 

Routines that prioritise oral hygiene were identified as essential to maintaining oral health behaviours for parents in this study. Regular water consumption was discussed by parents as important not only regarding fluoride exposure but also reduced sugar consumption. Just under half of the families included in this project live in non-metropolitan areas, where many houses rely on un-fluoridated rainwater. Routines that include enjoyable brushing time and reduced reliance on bottle use have previously been identified as critical to childhood oral health [77,81,84]. The use of fluoride varnish for children in this project provided an opportunity to further strengthen parental knowledge about the importance of brushing children’s teeth with fluoridated toothpaste for the prevention of ECC. Currently, fluoride varnish application is restricted to dental professionals in SA, despite its value in preventing ECC [85]. Researchers in New South Wales have successfully demonstrated that Indigenous dental assistants can effectively and safely apply fluoride varnish for children in schools; these findings have the potential to better oral health for Indigenous children across Australia [86]. 

### 4.3. Community-Level Influences 

While parents in our study identified positive dental experiences and compassionate practitioners as facilitators for dental service utilisation, these services are not easily accessible or available for all parents. Holistic health care and helpful community resources, such as ACCHS, AHW, and CaFHS nurses, were extremely important to parents in our project, especially those in regional areas with intermittent access to dental professionals. While many Indigenous peoples in Australia have priority access to dental care through the publicly funded system, various barriers prevent regular utilisation of mainstream preventive care [5,21,67,87]. Research with communities across Australia suggests that ACCHS reduce a number of barriers to preventive care, notably through increasing affordability and offering an appropriate model of care for Indigenous Australians [88]. Participants from a community-controlled midwifery program in Sydney [89] and a cardiac rehabilitation program in Perth [90] emphasised the flexible approach to care provided by ACCHS, as well as the availability of transportation, provision of informal child care, continuity of care, foundation of relationships and trust, and the utilisation of culturally appropriate information. Additionally, ACCHS works to counteract impacts of institutional racism that Indigenous peoples face when accessing mainstream health services [91]. Our findings support these notions; participants indicated they were more likely to access ACCHS because of existing supportive relationships and provision of holistic health care. 

Exploring alternative options for preventive oral health may increase accessibility for families and communities, such as the utilisation of AHWs in oral health promotion. Limited attention has been given to the potential for AHW involvement in oral health promotion in SA. Although the SADS have identified the importance of AHWs in oral health, utilising AHWs in a more official capacity could help address the challenges of maintaining oral health where services are not regularly available [92]. Training of community members as dental assistants has been effective in Alaskan Native communities and has been suggested for Canadian Inuit communities [93,94]. Community members in Alaska have been successful in bridging the gap between oral health professionals and communities, and providing more consistent and culturally competent care, especially in remote areas which have traditionally had sporadic access to dental care. In New South Wales, AHWs have been successful in facilitating oral health education programs for children and parents [95] and have self-identified their potential to facilitate similar programming for mothers during pregnancy [96]. Utilisation of AHWs for oral health provision builds on familiar and trusting relationships, which have been identified by both Indigenous parents and AHWs as imperative to service utilisation, attendance to appointments, and comprehensive identification of patient needs [97,98,99]. While the Indigenous oral health workforce is growing, there remains potential to strengthen Indigenous oral health in Australia by formalising the utilisation of AHWs in oral health promotion and dental assistance at a national level. 

The prioritisation of Indigenous partnerships in dental services would honour the significance of culturally determined understandings of illness and prevention and enhance cultural safety for Indigenous peoples accessing services [100,101]. Due to the fundamental gap in mainstream service provision, the focus on strengthening cultural competence among dental students and professionals has increased in recent years [102]. Culturally responsive engagement that acknowledges and works within the historical context and current experiences of Indigenous peoples would enhance the success of any oral health intervention [103].

Generational teaching, particularly from parents, grandparents, and elders, heavily influenced parent oral health beliefs and practices in our project. Previous research has demonstrated benefits to all aspects of wellness from Indigenous elder participation in educational and health-focused initiatives [104]. Elders play a key role in communication and knowledge transmission in communities and their opinions and advice are highly regarded on practical, spiritual, and moral grounds [104]. While some older participants from Tynan and colleagues felt they had missed opportunities to protect their own teeth, they exhibited concern for younger generations and wanted to ensure prevention awareness and better oral health outcomes for them [92]. Benefits have been observed not only for those involved in elder-informed projects, but to the elders themselves and the wider community, impacting cultural, environmental, and economic areas [98,105]. Elder involvement in community-based oral health prevention initiatives would help facilitate holistic oral health, beyond biomedical measures, and foster traditional connections and environments for children to thrive in [103].

### 4.4. Strengths and Limitations

This paper adds to the limited strength-based qualitative research on facilitators for improved Indigenous oral health in Australia. The utilisation of MI, importantly, respects Indigenous traditions of yarning and enabled participant engagement throughout the research process. The findings are unique in that they are framed within Fisher-Owens’ conceptual model for childhood oral health, which considers child-, family-, and community-level influences. While the unique directives for each interview may have influenced findings, the trusting relationships between the interviewer and participants and the MI skills exhibited by the interviewer enabled a free-flowing conversation and topics ranged widely between participants. The number of interviews and participants included in this analysis makes it unlikely that any significant issues were missed and the timing of interviews from 6–36 months of age gives good coverage of a variety of stages in first 2 years of life. A limitation of this paper is that MI is a behaviour change methodology—some findings may have manifested because of the intervention itself; nevertheless, participants identified these factors as benefits from participating in the project and facilitators for their children’s oral health. It is unlikely that the results discussed here would be the same from another interviewer; the variation in interviews across the staff conducting the MI was immense, hence the decision to include the interviews with the greatest depth and best quality to answer the research questions. Additionally, the dataset is from 2009–2013; therefore, findings do not consider the impact of oral health policy changes that have happened since data collection. Due to baseline recruitment occurring during pregnancy, the majority of all the interviews were conducted with mothers, despite the critical role of fathers, families, and communities in strengthening holistic oral health behaviours. 

## 5. Conclusions

The facilitators to better oral health of Indigenous children exist across child-, family-, and community-level influences. Prioritisation of community knowledge and experiences enables greater insight to factors that facilitate oral health and can contribute to the development of parent oral health understandings. Oral health professionals, researchers, and policy makers are encouraged to build upon facilitators identified by Indigenous peoples and centralise Indigenous leadership and partnerships in oral health service delivery and prevention programming. Future research should explore the perspectives of AHW regarding the incorporation of oral health programming through ACCHS, as well as dental professionals, regarding the feasibility of enhancing culturally accepted methods of care for Indigenous patients. Our recommendations from these findings include the following: (1) an exploration of AHW and elder participation in community oral health initiatives and mainstream dental services; (2) an inclusion of ACCHS representatives in mainstream oral health discussions and planning in South Australia; (3) an incorporation of child-level, family-level, and community-level facilitators, reflective of holistic understandings of health, to increase support for efficacious oral health programs. 

## Figures and Tables

**Table 1:** Categorisation of SEP measures.

Original Measures	Dichotomised	Categorisation
	Health care card status	
Yes	Yes	Low SEP
No	No	High SEP
	Maternal education	
No school	High school or less	Low SEP
Primary school		
High school	Trade or University	High SEP
Trade/TAFE		
University		
	Car ownership	
Yes	Yes	High SEP
No	No	Low SEP
	Difficulty paying AUD 100 dental bill	
Not hard at all	Minimal difficulty (Not hard at all, Not very hard)	High SEP
Not very hard		
A little bit hard	Some difficulty (A little bit hard, Very hard, Could not pay)	
Very hard		Low SEP
Could not pay		
Index of relative socioeconomic advantage and disadvantage (IRSAD)		
Decile 1–10	Decile 1–5	Low SEP
	Decile 6–10	High SEP

**Table 2:** Participant characteristics.

Measure	Overall Sample (*N* = 226) ^1^ *N* (%)
Health care card status	
Yes	176 (80.0%)
No	44 (20.0%)
Maternal education	
High school or less	151 (67.7%)
Trade or University	72 (32.3%)
Car ownership	
Yes	122 (54.7%)
No	101 (45.3%)
Difficulty paying AUD 100 dental bill	
Minimal difficulty	49 (22.0%)
Some difficulty	174 (78.0%)
IRSAD	
Decile 1–5	200 (90.9%)
Decile 6–10	20 (9.1%)
**Socioeconomic Position (SEP)**	
High SEP	63 (28.9%)
Low SEP	155 (71.1%)
**Mean Maternal Age in Years (SD)**	28.5 (6.65)

^1^ Note: Data not available for each participant in each category; where five measures were not available, socioeconomic position was not categorised.

## Data Availability

The data presented in this study are available upon reasonable request from the corresponding author. The data are not publicly available due to conditions of ethics approval.

## Supplementary Materials

The following supporting information can be downloaded at: https://www.mdpi.com/article/10.3390/ijerph19031150/s1, S1: COREQ (Consolidated criteria for Reporting Qualitative research) Checklist. S2: CONSORT 2010 checklist of information to include when reporting a randomised trial.
